# Supplementary material for: Circulating Citrate Is Associated with Liver Fibrosis in Nonalcoholic Fatty Liver Disease and Nonalcoholic Steatohepatitis
Source: Int J Mol Sci. 2023 Aug 28;24(17):13332. doi: 10.3390/ijms241713332 (PMC10487511; doi:10.3390/ijms241713332)
Supplement: Supplementary file 1 [file ijms-24-13332-s001.zip › ijms-2564492-supplementary.pdf]

Supplemental Table S1. Association of higher citrate with liver fibrosis stages in the NAFLD cohort (n=187).

Regression Models		Number of subjects (% of total)	Odds Ratio (95% CI)	*p*-value
Overall ordinal logistic regression analysis*†			1.41 (1.03 – 1.93)	0.03
Intermediate logistic regression analysis*	F0 vs. F1/F2/F3/F4	F1/F2/F3/F4: 138 (70.1)	1.75 (1.11 – 2.77)	0.02
	F0/F1 vs. F2/F3/F4	F2/F3/F4: 95 (48.2)	1.47 (1.01 – 2.12)	0.04
	F0/F1/F2 vs. F3/F4	F3/F4: 34 (18.4)	1.23 (0.80 – 1.90)	0.35
	F0/F1/F2/F3 vs. F4	F4: 15 (7.4)	1.15 (0.61 – 2.18)	0.66

*Multivariable models adjusted for age, gender, metabolic syndrome, and NAFLD Activity Score (NAS) (effect estimate of adjusted variables will be shared upon request). †z-score for the citrate level was used in the multivariable ordinal and logistic regression models. **Abbreviations:** CI, confidence interval; NAFLD, nonalcoholic fatty liver disease.

Supplemental Table S2. Association between pyruvate levels and liver fibrosis in the NAFLD cohort (n=187).

Variable	Model 1 (age, sex)		Model 2 (age, sex and metabolic syndrome)		Model 3 (age, sex, metabolic syndrome and NAS)	
	**β-coefficient (95% CI)**	***p*-value**	**β-coefficient (95% CI)**	***p*-value**	**β-coefficient (95% CI)**	***p*-value**
Pyruvate†	0.16 (-0.002 – 0.35)	0.05	0.12 (-0.05 – 0.30)	0.17	0.06 (-0.10 – 0.22)	0.45
Age	0.02 (0.01 – 0.03)	0.004	0.01 (-0.004 – 0.02)	0.16	0.02 (0.004 – 0.03)	0.01
Female	-0.30 (-0.66 – 0.07)	0.11	-0.32 (-0.68 – 0.05)	0.09	-0.46 (-0.79 – -0.14)	0.006
Metabolic syndrome			0.85 (0.47 – 1.22)	<0.001	0.70 (0.35 – 1.04)	<0.001
NAS					0.36 (0.25 – 0.47)	<0.001

†z-score for the serum pyruvate level and overall liver fibrosis (F0-F4) as a continuous variable were used in the multivariable regression models. **Abbreviations:** NAS, non-alcoholic fatty liver disease activity score. **Abbreviations:** CI, confidence interval; NAFLD, nonalcoholic fatty liver disease; NAS, NAFLD activity score.

Supplemental Table S3. Association between total ketone body levels and liver fibrosis in the NAFLD cohort (n=187).

Variable	Model 1 (age, sex)		Model 2 (age, sex and metabolic syndrome)		Model 3 (age, sex, metabolic syndrome and NAS)	
	β-coefficient (95% CI)	*p*-value	β-coefficient (95% CI)	*p*-value	β-coefficient (95% CI)	*p*-value
Ketone bodies†	0.09 (-0.09 – 0.26)	0.32	0.09 (-0.07 – 0.26)	0.28	0.14 (-0.005 – 0.29)	0.057
Age	0.02 (0.01 – 0.04)	0.002	0.01 (-0.004 – 0.02)	0.17	0.02 (0.005 – 0.03)	0.008
Female	-0.26 (-0.62 – 0.11)	0.16	-0.30 (-0.66 – 0.06)	0.10	-0.49 (-0.81 – 0.17)	0.003
Metabolic syndrome			0.90 (0.52 – 1.27)	<0.001	0.72 (0.39 – 1.06)	<0.001
NAS					0.39 (0.28 – 0.50)	<0.001

†z-score for the total ketone body level and overall liver fibrosis (F0-F4) as a continuous variable were used in the multivariable regression models. **Abbreviations:** NAS, non-alcoholic fatty liver disease activity score. **Abbreviations:** CI, confidence interval; NAFLD, nonalcoholic fatty liver disease; NAS, NAFLD activity score.
